# Evaluating Singapore’s CHAT Assessment Service by the World Mental Health Organisation (WHO) “Youth-Friendly” Health Services Framework

**DOI:** 10.3389/fpsyt.2019.00422

**Published:** 2019-06-20

**Authors:** Yi Ping Lee, Nur Khairunisa Binte Ngaiman, Lye Yin Poon, Hanisah Binte Abdul Jalil, Ming Hui Yap, Edimansyah Abdin, Mythily Subramaniam, Helen Lee, Swapna K. Verma

**Affiliations:** ^1^Early Psychosis Intervention Programme (EPIP), Institute of Mental Health, Singapore, Singapore; ^2^Singapore Polytechnic, Singapore, Singapore; ^3^Institute of Mental Health, Singapore, Singapore; ^4^Research Division, Institute of Mental Health, Singapore, Singapore

**Keywords:** Singapore, mental health, youth, help-seeking, service evaluation, service feedback

## Abstract

Young people experience high rates of mental health issues. However, many do not seek professional help. In order to encourage help-seeking behavior among young people, it is important to ensure that services are youth-friendly. This study aims to evaluate the Community Health Assessment Team (CHAT)’s mental health assessment service model using the World Health Organization (WHO) youth-friendly health service framework of accessibility, acceptability, and appropriateness (AAA), and to ascertain the extent to which the CHAT service model is youth-friendly. Three hundred young people aged 16–30 years, who had gone through CHAT mental health assessments, completed a 27-item questionnaire. Majority rated the items in the questionnaire favorably. Our results suggest that majority of the young people who accessed CHAT mental health assessment service found it to be youth-friendly.

## Introduction

Adolescence and young adulthood are critical stages in the developmental lifespan where mental health issues are most prevalent. Research has shown that one out of every four to five young people in the general population will suffer from at least one mental disorder (1). In a population-based survey of mental disorders in Singapore, it was found that 90% of all adult-onset mental illnesses emerged before the age of 29 years and the younger age group (18–34 years old) was more likely to develop major depressive disorder, bipolar disorder or a comorbid mental disorder (2).

Despite the high prevalence of mental health issues in young people, professional help-seeking behaviour remains low. In a study by Chong et al. (3), large treatment gaps were found in alcohol abuse (96.2%), followed by obsessive–compulsive disorder (89.8%) and alcohol dependence (88.3%). The same study also found that those who had an earlier age of onset were less likely to have treatment contact. In fact, only 31.8% of these young people sought help (3). Some of the barriers to help-seeking identified were lack of mental health literacy, stigma to mental illness, concerns about confidentiality and trust, availability of resources (e.g., time and cost), lack of accessibility of service (e.g., opening hours and location), and concerns about the characteristics of the provider (e.g., being judgmental) (4).

The World Health Organization (WHO) has developed a framework for “youth-friendly” services to address these help-seeking barriers among distressed young people (5, 6). The core foundation of the framework includes the triple A’s—“Accessible, Acceptable and Appropriate,” where services are as follows: free or at low cost, have convenient operating hours and location, and short waiting time (accessible); respectful, private, and confidential, promote information sharing, and have workers who are sensitive and skilled in working with young people (acceptable); and fulfill the needs of the young people at the point of service delivery or through referral linkages (appropriate).

The Community Health Assessment Team (CHAT) Singapore was set up in 2009 to improve the awareness of mental health issues in young people and increase accessibility of mental health services to young people by reducing the barriers to entry to specialist services often caused by stigma and logistical issues such as cost, location, and lack of know-how on navigating the national health system. CHAT endeavours to make its service youth-friendly in a number of ways.

CHAT offers free mental health assessments to young people aged 16–30 years old currently residing in Singapore. Young people who wish to access the mental health assessment are able to book an appointment online, *via* phone or walk-in. As CHAT assessments are conducted most days in a week (including weekends and after office hours), young people can choose a day and time that is suitable for them. All referrals for CHAT assessments are screened for presenting mental health concerns, risk issues, past psychiatric history, and outstanding forensic issues. Unless there is risk of harm to self or others, no parental consent is required to access CHAT mental health assessment, thereby ensuring confidentiality. Young people who come forward for assessments are not registered with any hospital system.

CHAT assessments are conducted in private rooms within the CHAT Hub, which is located centrally in Singapore in a building where many youth-related services and activities are found, and is easily accessible *via* public transport. The CHAT assessment team comprises mental healthcare professionals (psychiatrists and allied health professionals) specially trained to help young people distressed with mental health concerns. To put the young people at ease, a CHAT staff goes through what they should expect during the assessment process and assures them of confidentiality before the assessment session starts. Towards the end of each session, the CHAT staff will share clinical impression and discuss with the young people how best to manage their concerns and presenting issues (7).

As part of improving accessibility to specialized mental health services, CHAT also offers referral to and coordination with downstream counseling centers or public hospitals to young people who have undergone the assessment.

In 2012, Muir et al. published a paper that applied the WHO accessibility, acceptability, and appropriateness (AAA) framework to explore the extent to which the Australian National Youth Mental Health Foundation, also known as Headspace, is youth-friendly. Findings from the paper suggested that Headspace was successful in implementing an AAA youth-friendly service based on the WHO framework. This was achieved through the physical locations, access to public transport, easy referral processes, and affordability (accessibility); interior design, engagement of young people, privacy and confidentiality, the skills and approach of service providers, and information and control afforded to young people (acceptability); and the availability of comprehensive, multi-disciplinary services (appropriate).

Similarly, making reference to this comprehensive WHO’s framework would help elucidate the extent to which CHAT is successful in providing a youth-friendly mental health service while allowing for youth mental health services worldwide to be benchmarked using a common framework. In addition, as CHAT is the only service in Singapore that offers an assessment service for young people, the findings from this study will be invaluable in improving our service to young people in Singapore.

This paper aimed to evaluate the extent to which CHAT service is youth-friendly based on WHO AAA youth-friendly service framework by collecting quantitative data from young people who had accessed CHAT’s mental health assessment service.

## Methods

From 2014 to 2016, a total of 1,613 young people approached CHAT for help with mental health concerns and 978 underwent scheduled assessments at CHAT. These CHAT-assessed young people were consecutively approached for participation in this study. Of these, 300 young people agreed to participate.

Due to a lack of pre-existing validated measurement tools, a 27-item questionnaire based on WHO youth-friendly service framework of AAA was developed for use in this study. Items were further adapted to describe CHAT’s service. CHAT Ambassadors (i.e., young people who volunteered their time in working closely with CHAT for service improvement) were then approached to ensure that all key components were covered and all questions were phrased in a language that is clear and easy to understand.

Participants gave verbal informed consent, and those who agreed to participate completed the questionnaire in the waiting area of CHAT Hub after the mental health assessment. Participants took about 15 minutes to complete the anonymous questionnaire, which was self-administered.

The study was approved by the Institute of Mental Health’s Clinical Review Committee (CRC) and the relevant ethics committee—National Healthcare Group (NHG) Domain Specific Review Board. On accessibility, participants were asked about the ease of getting to CHAT Hub, the time it took getting to CHAT Hub, how easy it was to request for a mental health assessment, ease in finding an appointment that fitted their schedule, and the significance of having the assessment free of charge in their decision to come for the assessment. In the acceptability dimension, participants were asked to appraise the confidentiality and quality of the service received, the CHAT staff who delivered the service to them and the privacy of the assessment space. Lastly, on appropriateness, participants were asked on their satisfaction with the help options provided to them, whether they agreed with the treatment recommendation, and how helpful they found the coordination service to downstream referrals.

Several socio-demographic questions such as gender, age, race, occupation, and highest level of education were included in the questionnaire. Participants were also asked as to how they came to know about CHAT mental health assessment service.

### Statistical Analysis

Given the development of a 27-item questionnaire for use in this study, there was a secondary interest to explore the factor structure of the questionnaire. Sample size calculation was based on fulfilling this intent. There are several recommendations for sample size calculation for factor analysis, the most widely used rule of thumb uses the ratio of the number of subjects (*N*) to the number of items (*p*) and this varies from 3 to 10 (8). According to a simulation study conducted by Rouquette and Falissard (9), they found that a minimum of 300 subjects was generally acceptable when performing principal component analysis involving three-factor structures with 30 items within the scale. Hence, we decided on the sample size of 300 for this study. All statistical analyses were performed using SAS version 9.3 for Windows. Descriptive statistics were computed for the basic demographics. For the questionnaire, mean and standard deviations were calculated for continuous variables and frequencies and percentages for categorical variables. We compared the percentage differences of the responses across genders and age groups using chi-square test. All statistical significant tests were set at *p* value < 0.05.

## Results

Majority of the participants were female (74.0%, 222/300) and students (83.2%, 248/298), with a mean age of 21.4 years. More than half of them were already aware that CHAT is a youth mental health service before making contact with CHAT (62.2%, 186/299), and most of them made first contact with CHAT online (42.5%, 127/299). There were some significant differences between those who participated versus those who did not participate in the study in terms of their mean age: participants (*M* = 21.36, *SD* = 2.97) and non-participants (*M* = 19.67, *SD* = 2.95); *t*(947) = 8.19, *p* = 0.00; gender, *X* ^2^ (1, *N* = 978) = 11.03, *p* < 0.01; and employment status, *X* ^2^ (3, *N* = 978) = 16.99, *p* < 0.01 (**Table 1**).

**Table 1:** Socio-demographic characteristics of young people assessed by Community Health Assessment Team (CHAT).

Variable		Participants		Non-participants		*t*	*p*
		*N* = 300		*N* = 678			
		*M*	*SD*	*M*	*SD*		
Variable		n	%	n	%	X^2^	p
Age (in years)		21.36	2.97	19.67	2.95	8.19	0.00*
Gender						11.03	0.00*
Male		78	26.0	250	36.9		
Female		222	74.0	428	63.1		
Age group						–	–
16–21		180	60.0	488	72.0		
22–30		120	40.0	152	22.4		
Below 16		–	–	9	1.3		
Missing data		–	–	29	4.3		
Race						1.25	0.74
Chinese		210	70.5	461	68.0		
Malay		52	17.5	126	18.6		
Indian		18	6.0	51	7.5		
Others		18	6.0	35	5.2		
Missing data		–	–	5	0.7		
Employment status						16.99	0.00*
Student		248	83.2	529	78.0		
National Service (NS)		2	0.7	41	6.0		
Employed		29	9.7	75	11.1		
Unemployed		19	6.4	29	4.3		
Missing data		-	-	4	0.6		
Highest educational level						–	–
Degree		33	11.1	–	–		
Diploma		53	17.8	–	–		
Pre-university		38	12.8	–	–		
Secondary		136	45.5	–	–		
Primary		13	4.4	–	–		
Others		25	8.4	–	–		
First contact with CHAT was made *via*						–	–
Online		127	42.5	550	81.1		
Phone call		73	24.4	78	11.5		
Face to face at CHAT Hub		24	8	50	7.4		
Another concerned person		75	25.1	0	0		
Were they already aware that CHAT is a youth mental health service before making contact with CHAT?						–	–
Yes		186	62.2	–	–		
No		113	37.8	–	–		

All male Singaporean citizens and second-generation permanent residents are required to undergo a period of compulsory enlistment in National Service (NS) upon the age of 16 years and 6 months for 2 years.

*p < .01.

In terms of the reliability of the questionnaire used in this study, the Cronbach’s alpha coefficient for the total score was 0.724. Overall, responses from the questionnaire indicated that the participants found CHAT assessment service to be youth-friendly. Satisfaction rate with the treatment or help options offered was high, with 98.7% (296/300) indicating that they are somewhat or very satisfied with the help offered at the end of the assessment (**Table 2**). Further analyses were conducted to compare the percentage differences of the responses across genders and age groups, and no significant differences were observed. Factor analyses were not conducted as the data was extremely skewed as participants tended to report high scores across all items.

**Table 2:** Descriptive analysis of youth-friendly determinants (*n* = 300).

Variable	*N* (%)
**2.1 Accessibility**	
**It is easy to get to CHAT Hub.**	
Agree	284 (94.7)
Disagree	16 (5.3)
Missing data	0 (0.0)
**It takes 1 h or less to get to CHAT Hub.**	
Agree	252 (84.0)
Disagree	48 (16.0)
Missing data	0 (0.0)
**It is easy to request for CHAT mental health assessment.**	
Agree	286 (95.3)
Disagree	8 (2.7)
Missing data	6 (2.0)
**It is easy to get an appointment that fits with my schedule.**	
Agree	267 (91.4)
Disagree	25 (8.6)
Missing data	8 (2.7)
**The fact that CHAT assessment is free plays a significant part in my decision to come for the assessment.**	
Agree	289 (96.7)
Disagree	10 (3.3)
Missing data	1 (0.3)
**2.2 Acceptability**	
**I feel confident that the information I shared with CHAT will be confidential.**	
Agree	296 (98.7)
Disagree	4 (1.3)
Missing data	0 (0.0)
**In my first contact with CHAT, I find the CHAT staff to be friendly.**	
Agree	297 (99.0)
Disagree	2 (0.7)
Missing data	1 (0.3)
**The CHAT staff gave a clear explanation of what I can expect from the assessment.**	
Agree	297 (99.0)
Disagree	2 (0.7)
Missing data	1 (0.3)
**The waiting time between my first contact with CHAT and appointment given for CHAT assessment is acceptable.**	
Agree	296 (98.7)
Disagree	4 (1.3)
Missing data	0 (0.0)
**The SMS or phone call reminder of my CHAT assessment appointment is useful.**	
Agree	281 (93.7)
Disagree	18 (6.0)
Missing data	1 (0.3)
**Once I arrived at CHAT Hub for the assessment, I find the waiting time to see the assessment team not too long.**	
Agree	282 (94.0)
Disagree	18 (6.0)
Missing data	0 (0.0)
**The information sheet is helpful in assuring me of the confidentiality of the assessment.**	
Agree	263 (87.7)
Disagree	26 (8.7)
Missing data	11 (3.7)
**CHAT Hub offers a safe space for me to seek help for my mental health concerns.**	
Agree	297 (99.0)
Disagree	3 (1.0)
Missing data	0 (0.0)
**The environment in CHAT Hub allows me privacy to share information during the assessment.**	
Agree	293 (97.7)
Disagree	7 (2.3)
Missing data	0 (0.0)
**I find the assessment team approachable.**	
Agree	297 (99.0)
Disagree	3 (1.0)
Missing data	0 (0.0)
**During the assessment, I feel that the assessment team has a good understanding of my concerns.**	
Agree	298 (99.3)
Disagree	2 (0.7)
Missing data	0 (0.0)
**I am involved with the assessment team in making decisions on addressing my concerns.**	
Agree	295 (98.3)
Disagree	5 (1.7)
Missing data	0 (0.0)
**The assessment team is knowledgeable in addressing my concerns.**	
Agree	299 (99.7)
Disagree	1 (0.3)
Missing data	0 (0.0)
**The assessment team carried themselves professionally.**	
Agree	300 (100.0)
Disagree	0 (0.0)
Missing data	0 (0.0)
**I find the duration of the assessment session not too long.**	
Agree	237 (79.0)
Disagree	63 (21.0)
Missing data	0 (0.0)
**2.3 Appropriateness**	
**I do not need to repeat my concerns when I see the assessment team.**	
Agree	103 (34.3)
Disagree	196 (65.3)
Missing data	1 (0.3)
**I am satisfied with the treatment/help options offered towards the end of the assessment.**	
Agree	296 (98.7)
Disagree	4 (1.3)
Missing data	0 (0.0)
**Given my concerns, I agree with the recommendations given by the assessment team.**	
Agree	297 (99.0)
Disagree	3 (1.0)
Missing data	0 (0.0)
**It is helpful that CHAT staff arranges an appointment for me with the hospital or counselling service that I was referred to by CHAT.**	
Agree	259 (86.3)
Disagree	3 (1.0)
Missing data	38 (12.7)

Items in the questionnaire were presented on a Likert scale (1 = “strongly agree” to 5 = “strongly disagree”), and responses were dichotomized. Percentages correspond to the total number of participants in the study (n = 300).

### Accessibility

More than 90% indicated that CHAT assessment service was accessible in that they found it easy to get to CHAT Hub (94.7%) and to request for a mental health assessment (95.3%) and that it was easy to get an appointment that fit their schedule (91.4%). In addition, the assessment being free of charge made a significant impact on their decision to come for the assessment (96.7%) (**Table 2**, **2.1**).

### Acceptability

In most of the items under *acceptability* criteria, more than 90% of the participants indicated that CHAT assessment service was acceptable (**Table 2**, **2.2**). Ninety-eight point seven percent were confident that the information shared was kept confidential, and 99.0% found that CHAT Hub provided a safe and private space to share information. Wait time between the initial contact with CHAT and the scheduled CHAT assessment was not long and acceptable (98.7%), and 93.7% found the SMS reminder for their assessment useful. 99.0% of the participants found the assessment team to be approachable. More than 99.0% of them found the assessment team to have a good understanding of their issues and was knowledgeable and professional in addressing their concerns. They felt involved in making decisions to address their concerns (98.3%), and they were clear of what to expect from the assessment (99.0%).

That said, only 79.0% of the participants indicated that the duration of the assessment was not too long, and this was the lowest rated item in the acceptability criteria (**Table 2**, **2.2**).

### Appropriateness

Ninety-nine percent of the participants indicated that they agreed with the recommendations given by the assessment team, and 98.7% of them were satisfied with the help options offered. They also found it helpful that CHAT made the appointment to the referred help service on their behalves (86.3%) (**Table 2**, **2.3**).

However, only 34.3% indicated that they did not have to repeat their concerns when they saw the assessment team (**Table 2**, **2.3**), and this was the lowest rated item in the appropriateness criteria.

## Discussion

Two interesting findings emerged from this study, which highlights the challenge of implementing a youth-friendly service that is acceptable to young people and an opportunity for CHAT to relook into the assessment delivery. First, the duration of assessment was acceptable only to 79.0% of the participants, while the remaining 21.0% of participants found the duration of assessment to be long. While it seems intuitive that shortening the duration of assessment would help with improving so service aspect of acceptability, we need to ensure that doing so does not shortchange the quality of assessment. Despite having 21.0% of participants finding the duration of assessment to be long, 99.3% of participants in this study perceived the assessment team to have a good understanding of their concerns. In addition, 98.3% of participants liked that they were involved with the assessment team in making decisions to address their concerns. Time is required when we strive to achieve a comprehensive discussion of young people’s distress and concerns; explore their psychological, physical, social, and developmental needs (6); and, at the same time, ensure that young people feel empowered and involved in care planning during assessment. Inevitably, these may have led to a lengthy assessment process for the young people. Therein lies the challenge of implementing a youth-friendly service that is acceptable to young people.

Our second finding offers some insight to address this challenge. Specifically, 65.3% of participants may have to repeat their concerns when they saw the assessment team in CHAT. This suggests that duplication of information gathering may have happened at both the initial triage and subsequent assessment processes. Reviewing the way we deliver CHAT assessment may help to improve both the appropriateness and acceptability of CHAT assessment to young people. For example, better use of information gathered at the initial screening/triage by the assessment team may shorten the duration of assessment and avoid having the young people repeat their concerns. Upon meeting the young person for assessment, instead of asking the young person to share what brought him/her to CHAT again, the assessment team could then summarize the triage notes and use this to facilitate assessment of other areas concerning the young person’s mental health. Continuous training to ensure that youth mental health professionals stay competent is an important element for effective youth mental health services (10), and the inclusion of such engagement nuances during training for the assessment team is critical.

Research has shown that the use of technology plays a major role in the delivery of mental health services to youth in providing assessment, counseling through to treatment programs (11). In relooking the delivery of CHAT assessment, the team could also consider the use of technology to minimize repetition of concerns raised by young people and to enhance the overall assessment delivery.

As the findings attest, CHAT was generally successful in implementing a youth-friendly service consistent with the WHO “Accessible, Acceptable and Appropriate” framework. This was achieved through the ease of commuting to the physical location of CHAT Hub *via* public transport, an easy referral process, affordability (accessibility); having a safe environment in CHAT Hub, offering privacy and confidentiality, CHAT staff being professional yet friendly and approachable, offering information and giving choices to young people (acceptability); providing recommendations of treatment/help options fitting with the young people’s concerns and making timely referrals to these options (appropriate) (12). CHAT’s achievements are also due to ongoing efforts in engaging young people for their views and insights from the initial planning of CHAT service to the establishment of CHAT Ambassadors project. The CHAT Ambassadors project recruits young people on a yearly basis and actively engages them in CHAT’s service development and improvement. Studies have shown that consumer participation has the capacity to improve service quality, improve health outcomes for those involved, make services more responsive to consumers’ needs, and improve health outcomes of the wider population of consumers (12). In alignment with this principle, CHAT strives to continue efforts to engage young people in our service improvement so as to ensure that our service remains accessible, acceptable, appropriate, and friendly to young people in need of mental health support.

## Limitations

The quantitative nature of this study limited insights gained from participants’ perception of CHAT as an accessible, acceptable, and appropriate youth-friendly mental health service. Specifically, in-depth interviews with participants who indicated the duration of CHAT assessment was long and that they had to repeat their concerns when they met with the assessment team would shed further insight on whether these have negatively or positively impacted their experience with CHAT. The use of pre-determined criteria also limited the scope of this study to understand other factors necessary for the development of a youth relevant service from the young person’s perspective. Young people who consented to participate in the study differed from non-participants in that they were more likely to be older and of female gender. However, further analyses did not find significant differences in responses between the various age groups and across genders, and hence, the socio-demographic differences between the participants and non-participants may not have impacted the findings of the study significantly. Despite the detailed description of the WHO youth-friendly health service framework of accessibility, acceptability, and appropriateness, there are no validated measurement tools available. Thus, we had to develop our own questionnaire to measure youth-friendliness of our service. The lack of a well-validated questionnaire in this study limits the comparability of our results across settings and programs. Aligned with WHO’s move beyond youth-friendly to youth-responsive health services, future research in the development of validated measurements of youth-friendliness/youth-responsiveness will aid in future evaluation efforts by CHAT and other youth mental health services across the world.

## Ethics Statement

The protocol was approved by the NHG Domain Specific Review Board (DSRB).This study was carried out under the oversight of NHG Domain Specific Review Board and in accordance with the principles of Declaration of Helsinki. Participants gave oral informed consent and those who agreed to participate completed the questionnaire in the waiting area of CHAT Hub after the mental health assessment.

## Author Contributions

Conception, design of study, drafting article content—NN, LP, YL. Acquisition of data—HA, MY. Interpretation of data—YL, LP, NN, EA. Data analysis—EA. Critical revision of article content—SV, MS, YL, HL, NN, EA, LP. Approval of article content for submission—SV, MS, HL. Agree for accountability—YL, PY, NN, HA, YH, EA, MS, HL, SV.

## Funding

This research study was supported by the Singapore Ministry of Health’s National Medical Research Council under the Center Grant Program (NMRC/CG/004/2013). The funding source had no involvement in the study design, data collection, data analysis and interpretation. The funding body was not involved in the writing of the manuscript or the decision to submit the article for publication.

## Conflict of Interest Statement

The authors declare that the research was conducted in the absence of any commercial or financial relationships that could be construed as a potential conflict of interest.

## References

[1] Patel V Flisher AJ Hetrick S McGorry P Mental health of young people: a global public-health challenge. Lancet (2007) 369(9569):1302–13. 10.1016/S0140-6736(07)60368-7 17434406

[2] Chong SA Abdin E Vaingankar J Heng D Sherbourne C Yap M A population-based survey of mental disorders in Singapore. Ann Acad Med Singapore (2012) 41(2):49–66. 22498852

[3] Chong SA Abdin E Sherbourne C Vaingankar J Heng D Yap M Treatment gap in common mental disorders: the Singapore perspective. Epidemiol Psychiatr Sci (2012) 21(2):195–202. 10.1017/S2045796011000771 22789169

[4] Gulliver A Griffiths KM Christensen H Perceived barriers and facilitators to mental health help-seeking in young people: a systematic review. BMC Psychiatry (2010) 10:113. 10.1186/1471-244X-10-113 21192795 PMC3022639

[5] Muir K Powell A McDermott S ‘They don’t treat you like a virus’: youth-friendly lessons from the Australian National Youth Mental Health Foundation. Health Soc Care Community (2012) 20(2):181–9. 10.1111/j.1365-2524.2011.01029.x 21929697

[6] WHO Adolescent friendly health services. Geneva: World Health Organization Press (2002).

[7] Poon LY Tay E Lee YP Lee H Verma S Making in-roads across the youth mental health landscape in Singapore: the Community Health Assessment Team (CHAT). Early Interv Psychiatry (2014) 10(2):171–7. 10.1111/eip.12192 25277826

[8] MacCallum RC Widaman KF Zhang S Hong S Sample size in factor analysis. Psychol Methods (1999) 4(1):84–99. 10.1037//1082-989X.4.1.84

[9] Rouquette A Falissard B Sample size requirements for the internal validation of psychiatric scales. Int J Methods Psychiatr Res (2011) 20(4):235–49. 10.1002/mpr.352 PMC7549437 22020761

[10] Thomee S Malm D Christianson M Hurtig A Wiklund M Waenerlund A Challenges and strategies for sustaining youth-friendly health services - a qualitative study from the perspective of professionals at youth clinics in northern Sweden. Reprod Health (2016) 13:147. 10.1186/s12978-016-0261-6 28003025 PMC5178097

[11] Boydell KM Hodgins M Pignatiello A Teshima J Edwards H Willis D Using technology to deliver mental health services to children and youth: a scoping review. J Can Acad Child Adolesc Psychiatry (2014) 23(2):87–99. 24872824 PMC4032077

[12] Percy-Smith B ‘You think you know? …you have no idea’: youth participation in health policy development. Health Educ Res (2007) 22(6):879–94. 10.1093/her/cym032 17652346

